# Reproducibility of fetal global longitudinal strain measured with speckle tracking echocardiography using a fixed cardiac cycle

**DOI:** 10.1371/journal.pone.0354514

**Published:** 2026-07-31

**Authors:** Eline Meireson, Chantelle de Vet, Judith O. E. H. van Laar, Phaedra Mussche, Kristien Roelens, Noortje H. M. van Oostrum

**Affiliations:** 1 Department of Obstetrics and Gynecology, Ghent University Hospital, Ghent, Belgium; 2 Department of Human Structure and Repair, Ghent University, Ghent, Belgium; 3 Department of Obstetrics and Gynecology, Maxima Medical Center, Veldhoven, The Netherlands; 4 Department of Electrical Engineering, Eindhoven University of Technology, Eindhoven, The Netherlands; Hospital Sant Joan de Déu, SPAIN

## Abstract

Fetal speckle tracking echocardiography is an ultrasound-based technique used to assess myocardial velocity and deformation of the fetal heart. Despite its potential, the method has not yet been integrated into routine pregnancy care, partly due to concerns about inconsistent reproducibility. This study aimed to evaluate the intra- and inter-observer reproducibility of global longitudinal strain measurements derived from a fixed fetal cardiac cycle, using speckle tracking echocardiography.

Healthy women with singleton pregnancies were enrolled during the second trimester. From enrolment until delivery, four-chamber view clips of the fetal heart were acquired every four weeks. For intra-observer reproducibility, a single observer analyzed the same heart cycle in one DICOM clip twice for global longitudinal strain in the left and right ventricles, with a minimum interval of two weeks between assessments in a blinded manner. For inter-observer reproducibility, two independent observers analyzed the same cardiac cycle within one DICOM clip.

A total of 124 women were included, yielding 632 ultrasound clips. Intra-observer reproducibility was poor to moderate for global longitudinal strain for the right and left ventricles. Inter-observer reproducibility demonstrated moderate to good reproducibility for global longitudinal strain in both ventricles. The reproducibility was generally higher in the left ventricle than in the right, and the highest reproducibility was observed before 32 weeks of gestation.

In conclusion, speckle tracking echocardiography during pregnancy showed variable reproducibility of strain analysis when performed on a fixed cardiac cycle, with more consistent results in the left ventricle. These findings support the potential utility of fetal speckle tracking echocardiography, while highlighting the need for further refinement of reproducibility before clinical implementation.

## Introduction

Fetal speckle tracking echocardiography (fSTE) is a promising ultrasound technique in fetal cardiac assessment. The offline tool uses the speckles in the ultrasound image to measure the deformation and velocity values of the myocardial wall [1]. fSTE offers notable advantages compared to the commonly used ultrasound techniques to evaluate fetal heart function. Advantages include the relative angle independence during image acquisition and its non-invasive nature, requiring only a four-chamber view of the fetal heart [2–6]. fSTE has been proven to be feasible, and reference values for global longitudinal strain (GLS) in healthy singleton fetuses have been established [5–9].

Despite its promise, fSTE remains primarily confined to research settings. This limited clinical adoption is likely due to inter-vendor variability and inconsistent findings regarding both deformation measurements and reproducibility [5,10–16]. As a result, internationally accepted guidelines are still lacking, and fSTE has not yet been integrated into routine prenatal care.

A critical factor in the reproducibility of fSTE measurements is the adherence to a strict protocol of image acquisition and fSTE analysis. High-quality four-chamber ultrasound clips are essential, and several technical parameters, such as frame rate and region of interest, must be carefully optimized [17–20]. Acquiring suitable images is especially challenging in fSTE compared to adult STE due to the small size of the fetal heart, frequent fetal movements, and the increasing difficulty of imaging in later gestational stages [3,9,21–23]. Furthermore, fSTE analysis involves multiple manual steps within the software, each of which may influence the GLS measurements and contribute to the variability in the outcomes [18].

The available literature on the reproducibility of fSTE is limited and presents inconsistent findings [5,11–16,22,24–26]. Reported intra- and inter-observer reproducibility varies from poor to excellent [5,11–16,22,24–26]. These inconsistencies may be explained by the methodological differences or the fact that the reproducibility has been assessed at different stages of the fSTE analysis, rather than systematically evaluating each step. As a result, it remains unclear which components of the fSTE procedure are the main sources of variability.

To address existing gaps in knowledge regarding the reproducibility of fSTE, this study evaluates both intra- and inter-observer reproducibility using a standardized approach. In contrast to previous studies, reproducibility is assessed under controlled conditions by analyzing the same cardiac cycle within a single high-quality four-chamber ultrasound clip in healthy fetuses. This design enables a more precise assessment of variability attributable to the analysis process itself, independent of acquisition-related factors.

## Materials and methods

### Study population

This prospective cohort study was conducted at Máxima Medical Centre, a tertiary care facility located in Veldhoven, The Netherlands. The study protocol for the collection of ultrasound clips was described before by van Oostrum et. al.[9,27]. In short: the study included healthy pregnant women at 18  + 0 – 21  + 6  weeks of gestation, and followed them until delivery. Every four weeks, fetal heart ultrasound scans were performed. The exclusion criteria were the following: multiple pregnancies, insufficient understanding of the Dutch language, fetal arrhythmia, any suspicion of fetal congenital anomalies that might influence fetal cardiac function, pre-existing maternal hypertensive disease and autoimmune disease, including systemic lupus erythematosus or diabetes mellitus. Women who developed fetal growth restriction, hypertensive disease, or gestational diabetes after inclusion were excluded from the cohort. Furthermore, women who delivered a neonate with birth weight < 10^th^ percentile (corrected for gender and gestational age at birth) or with congenital or genetic abnormality were excluded from the cohort. The medical ethics committee of the Máxima Medical Centre, Veldhoven, The Netherlands, approved the study (NL64999.015.18). All participants provided oral and written informed consent. The recruitment period started on 22-05-2018 and ended at 31-04-2019. Anonymized data was accessed on the 18^th^ November 2024 and used for this study.

### Fetal Echocardiography and fSTE analysis

The fetal ultrasounds were performed using a Philips EPIQ 7 W ultrasound (Philips, Eindhoven, The Netherlands) by experienced cardiac sonographers, following a standardized protocol specifically designed for fetal heart echocardiography [27]. Ultrasound assessments were scheduled every 4 weeks from enrolment (18 weeks – 22 weeks) until delivery or up to 41 weeks of gestational age [27].

Offline fSTE analysis was conducted using dedicated fetal cardiac software (2D Cardiac Performance 1.2, TomTec Imaging Systems GmbH, Munich, Germany). The fSTE analysis was performed on stored DICOM clips, following a previously described standardized protocol for clip acquisition [27]. Three DICOM clips per ultrasound were stored.

The different steps of the fSTE analysis in this study were the following: 1) The DICOM clip with the highest possible frame rate, visibility of the atrioventricular valves, absence of outflow vessels, and visibility of the entire four-chamber view was identified out of the 3 stored DICOM clips.

**Fig 1:**
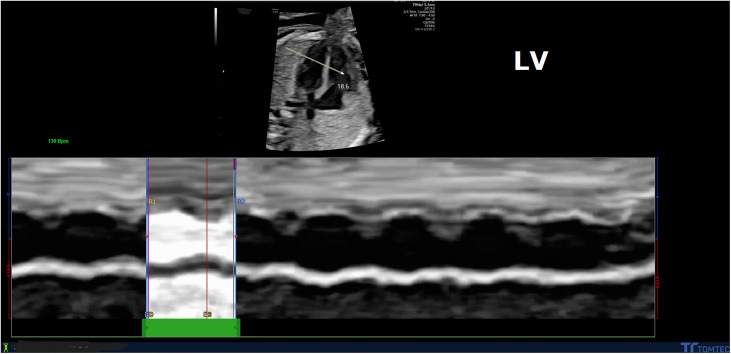
Step 2 of the fSTE analysis: selection of one heart cycle in M mode.

**Fig 2:**
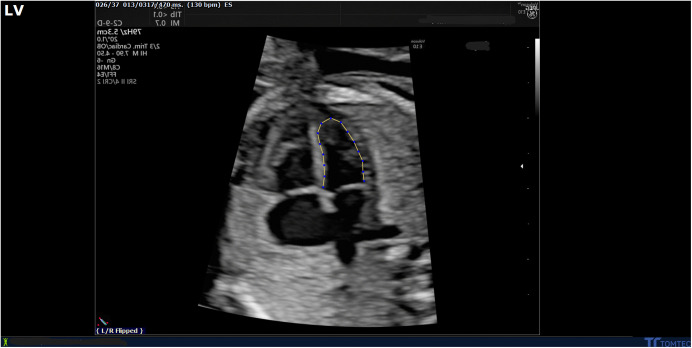
Step 3 of the fSTE analysis: delineation of the endocardial border in systole in the LV.

**Fig 3:**
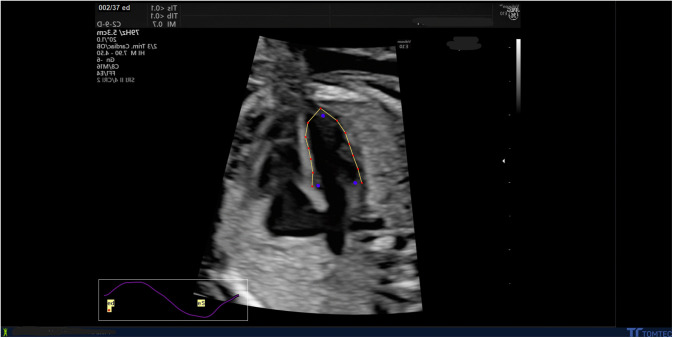
Step 4 of the fSTE analysis: delineation of the endocardial border in diastole in the LV.

**Fig 4:**
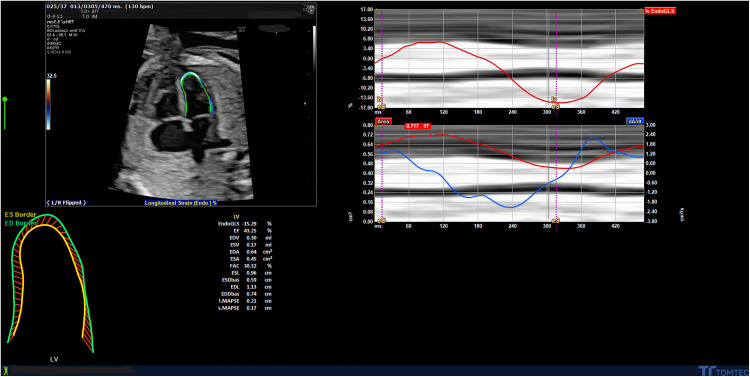
Step 5 of fSTE analysis: calculation of LV-GLS.

**Fig 5:**
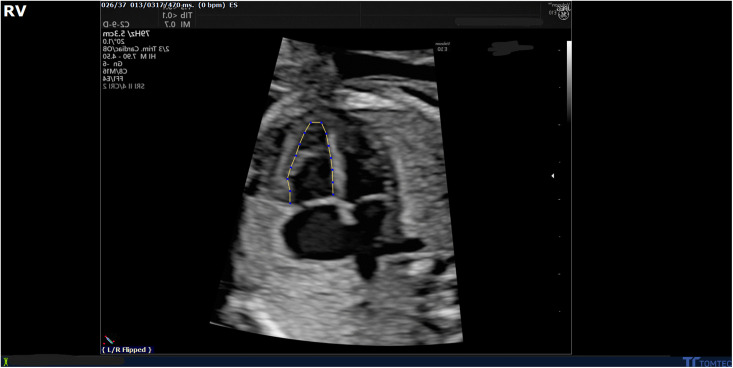
Step 6 of the fSTE analysis: delineation of the endocardial border of the RV.

2) Within this selected high-quality DICOM clip, 1 single cardiac cycle was selected. The M-mode and its corresponding R-wave functionality were used to identify the opening and closure of the atrioventricular valves, corresponding to one heart cycle (Fig 1).

3) Once one cardiac cycle was identified, the endocardial border of the left ventricle (LV) was defined by selecting three anatomical reference points (the junction of the lateral wall annulus, the junction of the septal wall annulus, and the apex of the heart). Based on these points, the software proposed an endocardial line in systole. If needed, this line could be manually adjusted (Fig 2).

4) After the endocardial border was set in systole, the software automatically generated the endocardial border in diastole. Manual corrections were again applied when the proposed contour did not accurately follow the endocardial wall (Fig 3).

5) After the completion of the endocardial wall identification in both systole and diastole, the software computed the LV-GLS (Fig 4).

6) Following the LV-GLS analysis, steps 3–5 were repeated for the right ventricle (RV), including the selection of reference points in the RV, contour verification in systole and diastole, and calculation of RV-GLS (Fig 5).

The offline fSTE analysis was performed by two fSTE experts (NvO and EM). NvO is an experienced specialist in fetal cardiac sonography with extensive expertise in fSTE analysis. EM is a PhD student whose research focuses on fSTE; at the start of her doctoral training, she received dedicated instruction in fSTE methodology. EM performed the fSTE analyses following the completion of NvO’s analyses.

To evaluate intra-observer reproducibility, NvO conducted the fSTE analysis twice, following the standardized protocol described above. The second analysis was performed in a blinded manner, starting from step 3 of the protocol, with a minimum interval of two weeks between both analyses. To assess inter-observer reproducibility, two observers (NvO and EM) independently performed GLS measurements, blinded to each other’s outcome measures, and with a minimum interval of two weeks between both analyses. For the inter-observer reproducibility analysis, the second observer (EM) used the cardiac DICOM clips and heart cycles as identified by the first observer (in steps 1 and 2). fSTE analysis was performed by the second observer (EM) from steps 3 onwards. The second observer was blinded to the GLS values of the first observer. For this analysis, one ultrasound clip per participant was selected within a gestational age range of 23–30 weeks. With this protocol, we ensured the same DICOM clip and heart cycle were used, prohibiting differences in deformation values due to measurements in different heart cycles. The obtained GLS measurements of the three analyses were used to calculate the intra- and inter-observer reproducibility.

### Statistical analysis

Statistical analyses were conducted using RStudio (version 2025.05.1+513). Continuous variables were reported as means and standard deviations (±SD). To evaluate the reliability of measurement agreement, the intraclass correlation coefficient (ICC) was used. The clinical interpretation of ICC values was categorized as follows: values > 0.9 indicate excellent reliability, 0.75–0.90 good reliability, 0.50–0.75 moderate reliability, and values below 0.50 reflect poor reliability [5,28].

First, **intra-observer reproducibility** was assessed using the dataset containing LV and RV-GLS values calculated twice by the first observer (NvO). ICC values and their corresponding 95% confidence intervals (CIs) were estimated using a linear mixed-effects model (LMM), which accounted for both within-subject and between-subject variance components. Separate models were constructed to assess variability in LV- and RV-GLS measurements. Gestational age was included as a fixed effect, while patient study ID and analysis number (analysis one or two) within each patient were modelled as random intercepts. The ICC was calculated as the proportion of total variance attributable to between-subject variability. This approach corresponds to a single-measure, one-way random-effects ICC (ICC(1,1)), reflecting absolute agreement between repeated measurements. Bland–Altman plots were made to visualize the agreement of the overall GLS in both the LV and RV between analyses one and two, illustrating the mean difference along with the upper and lower limits of agreement. Bland–Altman plots were constructed for descriptive assessment of agreement. Given the presence of repeated measurements per subject, these analyses do not fully account for within-subject correlation and should be interpreted accordingly. Afterward, a paired t-test was performed to assess systematic bias between GLS values obtained from analyses one and two. A non-significant result (p > 0.05) indicates no statistically meaningful difference, suggesting comparable outcomes across both analyses.

In a sub-analysis, the intra-observer reproducibility was further evaluated across specific gestational age categories using the ICC function in RStudio, providing the ICC value along with its associated 95% confidence intervals. The following gestational age categories were created: 18–22, 23–27, 28–32, 33–37, and 38–41 weeks. Bland–Altman plots were generated for LV- and RV-GLS across these gestational age categories. These plots were used to visualize the agreement of the GLS measurements between analyses one and two, illustrating the mean difference along with the upper and lower limits of agreement per gestational age (GA) group. These plots were made for descriptives only. A paired t-test was performed to assess systematic bias between GLS values obtained from analyses one and two. A non-significant result (p > 0.05) indicated no statistically meaningful difference, suggesting comparable outcomes across both analyses.

Secondly, the **inter-observer reproducibility** was assessed. A sample size calculation was performed to determine the number of clips needed to be evaluated [29,30]. The threshold was set at 0.60 [25], expected reliability at 0.75, statistical power at 80%, and significance level at 0.05. Only independent data were used, with one DICOM clip per patient. The selected DICOM clips originated from gestational age categories that showed the highest reproducibility in the intra-observer sub-analysis. After the selection of the DICOM clips needed for the analysis, the same statistical methods as used for the intra-observer reproducibility analyses were applied, including an LMM, Bland-Altman plots, and paired t-tests.

## Results

The cohort's study characteristics have been previously described [9]. In brief, a total of 632 clips from 124 patients were collected between 18 and 41 weeks GA. The mean maternal body mass index was 23.89 kg/m² (±4.28). The mean frame rate of the ultrasound clips was 87.20 frames per second (fps) (±20.90). The mean LV- and RV-GLS were respectively −21.26% (±7.02) and −19.05% (±5.46).

### Intra-observer reproducibility

Table 1 summarizes the ICCs, and 95% CIs, of the LV- and RV-GLS (GA 18–41 weeks). The p-values from the paired t-test are both non-significant (p > 0.05) (Table 1).

**Table 1:** Intraclass coefficients (ICCs) for LV-and RV-GLS, along with their corresponding 95% confidence intervals (CIs), and p-value from the paired t-test.

	ICC	95% CI	p-value
LV GLS	0.651	[0.582 – 0.712]	0.435
RV GLS	0.460	[0.386 – 0.528]	0.199

**Fig 6:**
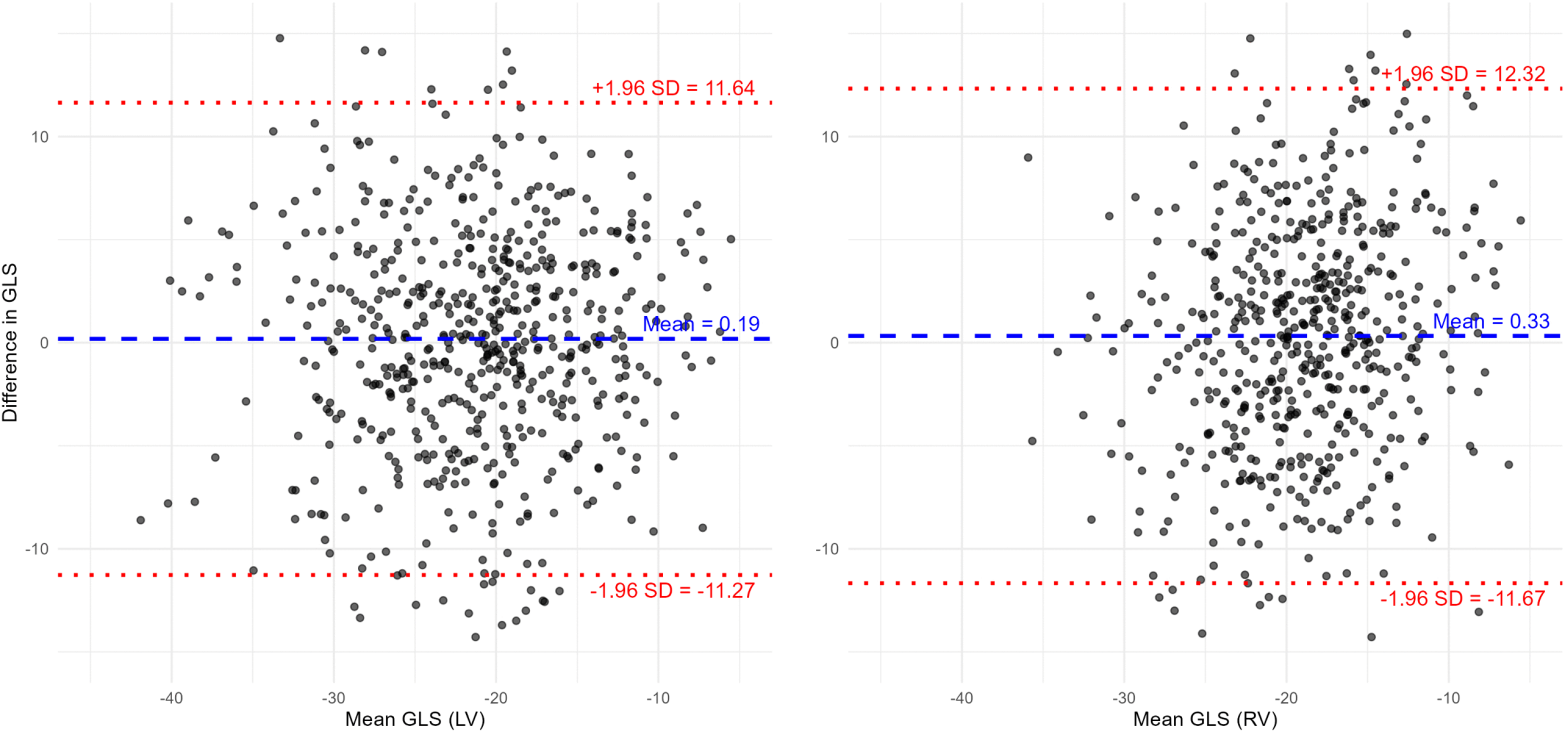
Bland-Altman plots (intra-observer) of the overall LV-and RV-GLS (GA ranging from 18 to 41 weeks).

The Bland-Altman plots of the overall LV-and RV-GLS (GA 18–41 weeks) of the intra-observer analysis are shown in Fig 6. The mean difference of LV-GLS was 0.19, with a lower and upper limit of −11.27% and 11.64%. The mean difference of RV-GLS was 0.33%, with a lower and upper limit of −11.67% and 12.32%.

Table 2 summarizes the ICCs of the GLS of the LV and RV at specific GA categories as well as the p-value from the paired t-test.

**Table 2:** Intraclass coefficient of LV-and RV-(GLS), along with their 95% confidence interval (CI) and p-value from the paired t-test, per GA category.

GA (weeks)	Clips (n)	LV-GLS			RV-GLS		
		ICC	95% CI	p-value	ICC	95% CI	p-value
18-22	128	0.687	[0.584-0.769]	0.093	0.379	[0.217-0.52]	0.721
23-27	184	0.736	[0.661-0.797]	0.188	0.505	[0.389-0.606]	0.437
28-32	139	0.647	[0.539-0.734]	0.728	0.518	[0.381-0.633]	0.362
33-37	109	0.405	[0.235-0.551]	0.246	0.356	[0.169-0.519]	0.688
38-41	46	0.633	[0.422-0.779]	0.45	0.504	[0.223-0.707]	0.715

**Fig 7:**
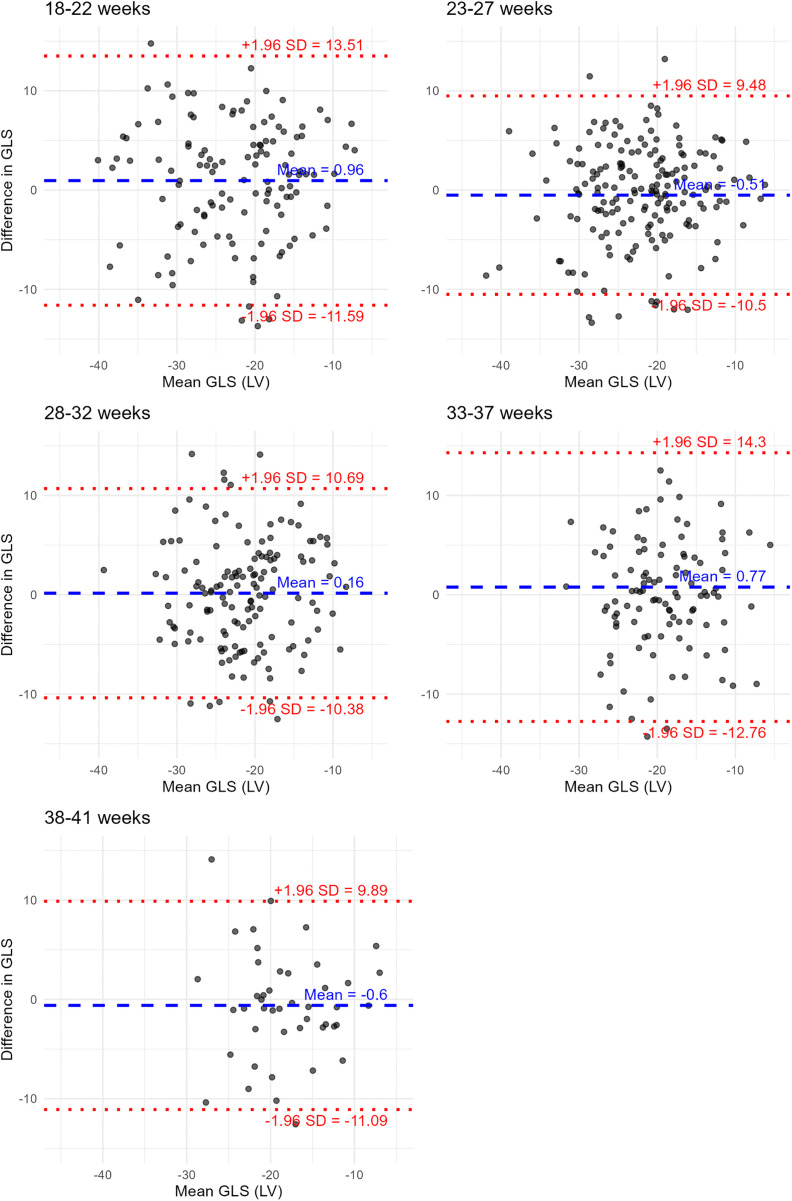
Bland-Altman plots (intra-observer) of the LV-GLS across different gestational age categories (18-22 weeks, 23-32 weeks, 33-37 weeks, 38-41 weeks).

The LV-GLS Bland-Altman plots performed across the gestational age categories of the intra-observer sub-analysis showed the narrowest spread within the 23–27 weeks group (Fig 7). Mean differences across all categories were close to zero, with the smallest observed in the 28–32 weeks group (mean difference = 0.16%).

**Fig 8:**
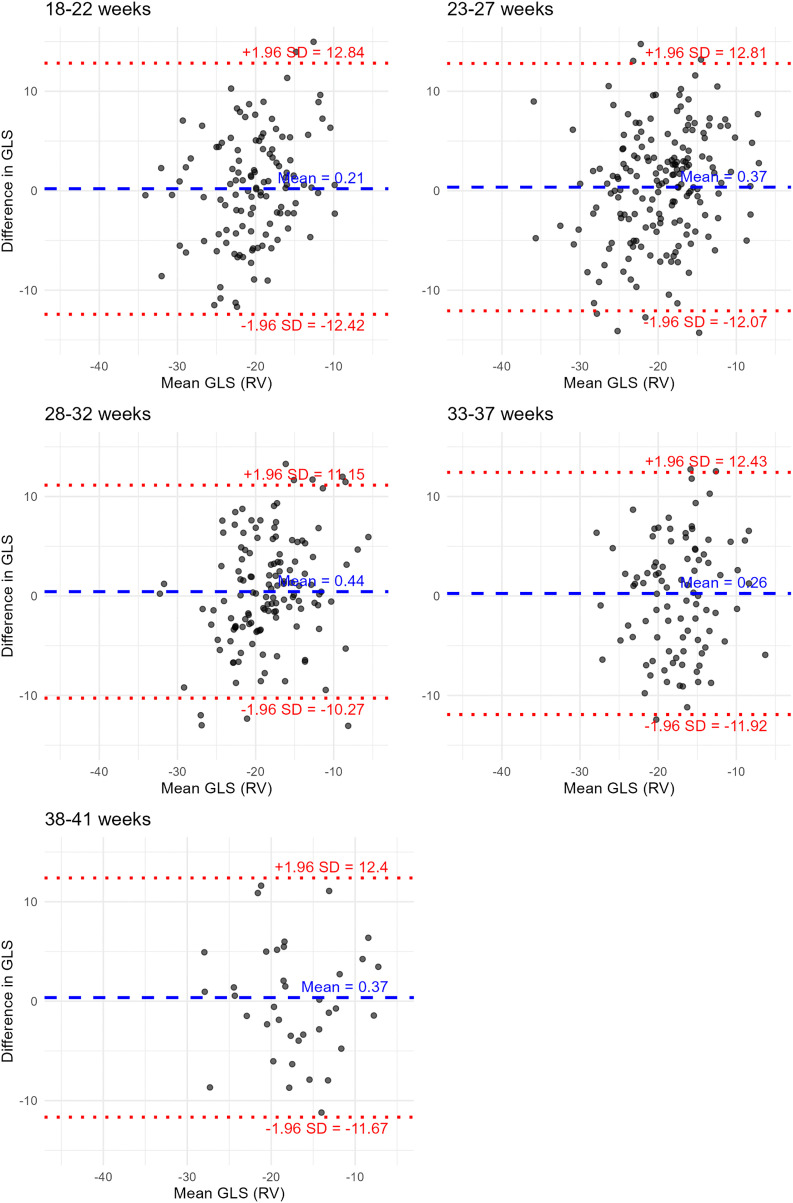
Bland -Altman plots (intra-observer) of RV-GLS across different GA categories (18-22 weeks, 23-32 weeks, 33-37 weeks, 38-41 weeks).

The RV-GLS Bland-Altman plots performed across the gestational age categories of the intra-observer sub-analysis demonstrated the narrowest spread within the 18–22 weeks and 23–27 weeks group (Fig 8). Mean differences across all categories were close to zero, with the smallest observed in the 18–22 weeks group (mean difference = 0.21%).

### Inter-observer reproducibility

The sample size calculation indicated that 102 independent ultrasound clips were required for the inter-observer reproducibility analysis. We selected of every participant 1 DICOM clip (124 clips), originating from the gestational ages of 23–30 weeks, with the majority (44 clips) performed at 27 weeks GA. Table 3 shows the ICCs calculated from an LMM along with their corresponding 95% CI, and their p-values from the paired t-test for the GLS values of both ventricles.

**Table 3:** Intraclass correlation coefficients (ICCs) for the LV-and RV-GLS, along with their corresponding 95% confidence intervals (CI), and p-value from the paired t-test.

	ICC	95% CI	p-value
LV GLS	0.871	[0.792 – 0.921]	0.589
RV GLS	0.662	[0.483 – 0.780]	0.0008

**Fig 9:**
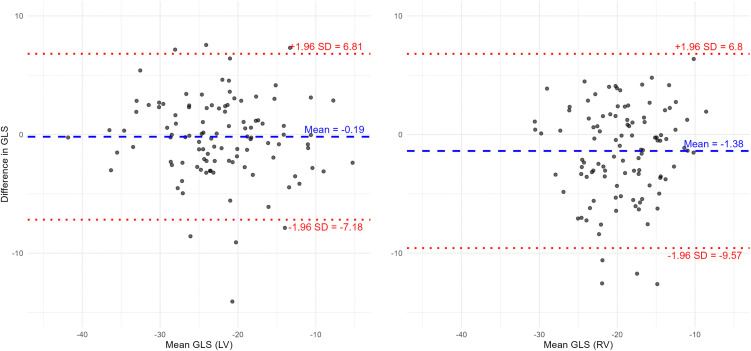
Bland-Altman plots of inter-observer differences in LV-and RV_GLS (GA ranging from 23 to 30 weeks).

The Bland-Altman plots of the LV-and RV-GLS (GA 23–30 weeks) of the inter-observer analysis are shown in Fig 9. The mean difference in LV-GLS is −0.19%, compared to −1.38% for RV-GLS.

## Discussion

This study specifically assesses the intra- and inter-observer reproducibility of fetal GLS derived from the same heart cycle within a single DICOM clip analyzed using fSTE with a standardized approach. Concerning the intra-observer reproducibility, our findings demonstrated moderate reproducibility for LV-GLS and poor reproducibility for RV-GLS. Importantly, the intra-observer reproducibility of both LV- and RV-GLS varied across gestational ages, with the highest ICC values reported before 32 weeks of gestation. Regarding inter-observer reproducibility, good reproducibility was shown for LV-GLS, and moderate reproducibility for RV-GLS.

Although several studies have reported on the intra- and inter-reproducibility of LV- and RV-GLS before [5,11,12,14–16,22,25,26], the results are difficult to compare and interpret due to several factors:

### Statistical methodology

Different studies have employed various statistical approaches to assess reproducibility, such as ICC, Lin’s Concordance Correlation Coefficient (CCC), Bland-Altman plots, Pearson’s correlation coefficient (r), and Cronbach’s alpha [5,11,12,14–16,22,26]. Among these, ICC is widely recognized as the most appropriate method for evaluating reproducibility in studies involving multiple raters, repeated measurements, and continuous outcomes [31,32]. Bland-Altman plots complement ICCs by visually representing agreement and identifying potential systematic bias [33]. Therefore, our study applied both ICC and Bland-Altman plots to assess reproducibility.

To date, eight studies have reported ICC values for LV- and RV-GLS reproducibility using fSTE [5,12,14–16,22,24,26]. The ICC values of these studies are summarized in Table 4. Reported ICC values for LV-GLS intra-observer reproducibility ranged from 0.35 [24] to 0.93 [12], aligning with our findings. Inter-observer ICCs for LV-GLS ranged from 0.44 [14] to 0.91 [22], also consistent with our results. For RV-GLS, intra-observer ICCs ranged from 0.21 [24] to 0.89 [12,16] and inter-observer ICCs from 0.28 [24] to 0.89 [26], both in line with our findings.

**Table 4:** Overview of published articles reporting on intra- and inter-observer reproducibility using Intraclass correlation coefficients (ICC) and their 95% confidence interval (CI) of LV-and RV-GLS.

Article	ICC LV GLS [95% CI]		ICC RV GLS [95% CI]		ICC categories used in the study
	Intra-observer	Inter-observer	Intra-observer	Inter-observer	
Our study	0.65[0.58- 0.71]	0.87[0.79- 0.92]	0.46[0.39- 0.53]	0.66[0.48- 0.77]	<0.50 poor 0.50-0.75 moderate 0.75-0.90 good >0.90 excellent
Kapusta et al., 2012 [14]	0.60*	0.44*	0.84*	0.68*	<0.40 poor 0.40-0.60 moderate 0.60-0.80 good >0.80 excellent
Crispi et al., 2012 [12]	0.93 [0.88- 0.96]	0.89 [0.81- 0.93]	0.89 [0.82- 0.93]	0.87 [0.79- 0.93]	No categories described
Kapusta et al., 2013 [22]	0.83*	0.91*	0.58*	0.85*	<0.40 poor 0.40-0.60 moderate 0.60-0.80 good >0.80 excellent
Enzensberger et al, 2017 [26]^$^	–	0.890*	–	0.892*	0.7-0.80 good >0.80 strong
Huntley et al., 2021 [15]	0.77[0.57-0.91]	0.65[0.43–0.79]	0.44[0.32–0.68]	0.58[0.32–0.74]	0.50-0.60 low 0.7-0.80 good >0.80 strong
Hu et al., 2024 [16]	0.78 [0.53- 0.91]	0.66 [0.33- 0.85]	0.89 [0.74- 0.96]	0.84 [0.64- 0.93]	<0.40 poor 0.40-0.75 moderate >0.75 good
Nichting et al., 2024 [5]	0.75[0.56-0.86]	0.67[0.45-0.82]	0.80[0.64-0.89]	0.75[0.57-0.86]	<0.50 poor 0.50-0.75 moderate 0.75-0.90 good >0.90 excellent
Mlodawski et al., 2025 [24]	0.35 [0.01-0.61]	0.5 [0.27-0.68]	0.21 [0.78-0.88]	0.28 [0.02-0.51]	<0.40 poor

*No 95% confidence interval (CI) was mentioned in the article, ^$^the inter-observer reproducibility was calculated in this study with Cronbach’s alpha and therefore not mentioned in the Table.

Three of the eight studies did not report the 95% CI for their ICC estimates [14,22,26]. According to the guideline for selecting and reporting the ICC for reproducibility analysis, reproducibility should be reported by specifying the software used for analysis, along with the ICC estimate and its 95% CI [31]. Without the 95% CI, the stability and precision of the ICC values cannot be assessed [14,22,26].

Importantly, there are no universally accepted thresholds for interpreting ICC values [31]. Classification criteria varied across studies (Table 4), with definitions of “poor” ICC ranging from <0.4 to <0.6, and “good” ICC from >0.6 to >0.95 [15,22,25]. Due to this inconsistency, only the raw ICC values can be meaningfully compared. Nevertheless, we encourage researchers to adopt consistent ICC classification criteria when the same methodology is used to enhance comparability and interpretability. Across studies [5,12,14–16,22,26], both intra- and inter-observer reproducibility were generally higher for LV-GLS than for RV-GLS [12,15,22,24], likely due to the more complex delineation of the RV endocardial border in fSTE software, particularly influenced by anatomical features such as the moderator band [34]. It is important to note that a low ICC does not necessarily indicate poor reliability. It may also result from low variability among subjects, a small sample size, or a limited number of raters [31].

Most studies reported higher intra-observer reproducibility [5,12,14–16]. Remarkably, our study found higher inter- than intra-observer reproducibility, in line with two other studies [22,24]. We suggest that the reason for a higher inter-observer reproducibility in our study could be the following: the inter-observer reproducibility was assessed with clips (GA ±27 weeks), which showed the highest reproducibility in the intra-observer analysis

Bland-Altman plots of the inter-observer reproducibility were provided by the studies of Crispi et al.[12], Hu et al.[16], Huntley et al.[15], and Nichting et al.[5], showing mean differences in the LV-GLS ranging from −4.48 [15] to 0.3 [12] and in the RV-GLS from −4.0 [16] and −0.7 [12]. Our results are comparable, with a mean difference of −0.19 in LV-GLS and −1.38 in the RV-GLS. Interestingly, our upper and lower limits showed the narrowest spread compared to the other studies. Bland-Altman plots evaluating intra-observer reproducibility were only provided in our study and that of Huntley et al.[15]. These showed mean differences of 0.19 and 6.42 for LV-GLS, and 0.33 and 4.89 for RV-GLS, respectively [15]. A mean difference closer to zero indicates greater agreement between measurements, suggesting higher reproducibility and consistency across observers or repeated analyses [33]. However, despite these small mean differences and only poor to moderate ICC values, the limits of agreement in our intra-observer study remained relatively wide (approximately ± −11–12%). Indicating substantial variability between repeated measurements. Consequently, individual GLS measurements may not be interchangeable, as repeated assessments can differ considerably. From a clinical perspective, small differences should therefore be interpreted with caution, as they may reflect measurement variability rather than true change. These findings highlight the importance of evaluating both consistency (ICC) and absolute agreement (Bland–Altman analysis) when assessing reproducibility.

### fSTE analysis step

Previous studies [5,11–16,22,26] have not specified which specific step of the fSTE analysis was selected for their reanalysis, leaving uncertainty about the comparability of the study results. As mentioned before, fSTE analysis requires manual adjustments in each step. Depending on the fSTE step used for the reproducibility measurements, outcomes might be different and variable. Our study is the first to evaluate GLS reproducibility using a fixed heart cycle within a single DICOM clip and to explicitly define and report the specific analysis step selected for reanalysis. This approach enhances methodological transparency and allows for a more accurate assessment of measurement reliability and the ability to reproduce the study. We encourage researchers to describe the methodology of their intra- and inter-reproducibility analysis in more detail. Specifically, describing the exact step in the fSTE analysis that was reanalyzed. This would lead to more transparency and the possibility of comparing the results.

### Sample size and gestational age

The number of included clips in the reproducibility analyses across previous studies varies considerably, ranging from 10 to 56 (Table 5) [5,11–16,22,24–26]. Based on the approaches described by Walter et al.[30] and Bonnett [35], a formal sample size calculation for assessing inter-observer reproducibility was performed for this study. This approach ensured adequate statistical power and strengthened the reliability of our findings [32]. We determined that a minimum of 102 clips was required to ensure a robust analysis of reproducibility using ICC as the reliability metric. This number is in great contrast with the included clips of previously published studies and makes its reliability questionable. Even more, other studies reanalyzed a random number of clips available from their cohort, without performing a sample size calculation [5,14–16,22].

**Table 5:** Characteristics of published articles analyzing the intra- and inter-observer reproducibility.

Article	Number of clips included	Ultrasound device	STE software	GA (weeks)	Frame rate criteria	Angle of insonation
Our study	632* or 124**	Philips Epiq W7	TomTec 2D Cardiac Performance 1.2 software	18-41	The highest possible	Not considered
Kapusta et al., 2012 [14]	12	Vivid I digital ultrasound scanner (GE)	EchoPac (GE)	20-24	≥ 90 Hz	Apical or basal
Crispi et al, 2012 [12]	56	Vivid I digital ultrasound scanner (GE)	EchoPac (GE)	23-40	>70Hz	Apical or basal
Kapusta et al., 2013 [22]	10	Vivid I digital ultrasound scanner (GE)	EchoPac (GE)	30-34	≥ 90 Hz	Apical or basal
Enzensberger et al.2017 [26]	10	Toshiba Medical Systems Corporation, Otawara, Tochigi, Japan	TestDriver software (Toshiba Medical Systems Corporation, Japan)	18-36	>60Hz	Apical
Huntley et al., 2021 [15]	14* or 50**	GE Voluson E10	FetalHQ software	20-38	≥ 80 Hz	Perpendicular or tangential
Hu et al., 2024 [16]	20	GE Voluson E10	FetalHQ software	20-41	≥ 80 Hz	Not described
Nichting et al., 2024 [5]	37	Philips Epiq W7	TomTec 2D Cardiac Performance 1.2 software	16	≥ 80 Hz	Not described
Mlodawski et al., 2025 [24]	34*, 53**	GE Volusion Expert 22	FetalHQ software	19-23	The highest possible	Perpendicular

* intra-observer reproducibility, **inter-observer reproducibility

Concerning the gestational age, our study observed variable reproducibility of the LV- and RV-GLS values between the gestational age categories. The highest intra-observer reproducibility was observed at 23–27 weeks for LV-GLS (ICC = 0.736; 95%CI [0.66−0.]) and at 28–32 weeks for RV-GLS (ICC = 0.518; 95%CI[0.381–0.633]). Lower ICC values were noted in later gestational stages, likely reflecting the increased difficulty of fetal cardiac imaging in the third trimester [36].

In previous studies [5,12,15,16,26], intra- and inter-observer reproducibility analyses were conducted using clips obtained across a wide range in gestational age(16–40 weeks), without accounting for gestational age in the analysis.

Kapusta et al. evaluated the intra-observer reproducibility in the second (20–24 weeks) and third trimester (30–34 weeks) [14,22]. Their reported ICC for LV-GLS in the second trimester was 0.60, slightly lower than our ICC value of 0.687 at 18–22 weeks and notably lower than our ICC value of 0.736 at 23–27 weeks. Interestingly, their ICC for RV-GLS was substantially higher (0.84) compared to our values of 0.379 at 18–22 weeks or 0.505 at 23–27 weeks of gestation. This is remarkable, since the delineation of the endocardial border of the RV is harder than in the LV [34]. In the third trimester, Kapusta et al.[22] reported ICC values of 0.83 for LV-GLS, which is notably higher than our ICC values of 0.647 at 28–32 weeks and 0.405 at 33–37 weeks. Similarly, for RV-GLS, their reported ICC of 0.58 is higher compared to our ICC values of 0.518 at 28–32 weeks and 0.356 at 33–37 weeks of gestation [14,22]. Despite the known challenges of cardiac imaging in the third trimester, Kapusta et al.[22] reported notably high ICC values. However, their reproducibility analysis was based on a small sample,12 clips in the second trimester and 10 in the third [14,22]. In contrast, our study included a substantially larger dataset, with 128 clips re-evaluated at 18–22 weeks, 184 at 23–27 weeks, 139 at 28–32 weeks, and 109 at 33–37 weeks.

### Technical characteristics

Other factors influencing the comparability of the intra- and inter-observer reproducibility analyses are the technical characteristics of the fSTE, particularly the image quality in each study. Image quality depends on factors such as acquisition technique, gestational age, and ultrasound settings (e.g., frame rate) (Table 5) [4,11–16,22,25,26]. While these technical aspects do not directly influence the reproducibility outcomes within a single study, it is important to report them to facilitate meaningful comparisons across studies.

Another factor influencing the comparability of results is the inter-vendor variability. The studies mentioned above used different types of STE software: TomTec, FetalHQ, and EchoPac (Table 5) [5,14–16,22]. However, the study by Di Tonto et al. has shown promising reproducibility between TomTec and FetalHQ software [25], suggesting that harmonization across vendors may be achievable with further validation.

In our study, the angle of insonation was not considered during image acquisition, based on prior evidence suggesting the software's relative angle independence [4]. However, the influence of insonation angle remains debated, as several studies have reported that variations in angle, as well as differences in frame rate, may affect tracking performance and reproducibility [12,14,15,22,24,26].

### Limitations

A few limitations in our study should be acknowledged. First, the quiver tool provided in the TomTec software was not employed during the fSTE analysis by the first observer and was therefore also omitted by the second observer. This may have influenced the precision of myocardial tracking and GLS measurements. However, there has been no difference in reproducibility shown using the quiver tool, only in the velocity of the analysis [15,37].

Second, the inter-observer reproducibility of the RV-GLS showed significant mean differences (paired t-test, *p* < 0.05; Bland-Altman mean difference of −1.46). This suggests the presence of systematic bias, where one observer may consistently overestimate GLS values relative to the other. While ICC values capture relative consistency, they do not account for absolute mean differences illustrated by the Bland-Altman plot, limiting clinical interchangeability. Previous studies by Nichting et al.[5] and Sacco et al.[11], also included a paired t-test to assess potential systematic bias, but reported *p*-values above 0.05, indicating no significant bias between observers.

Third, for the inter-observer reproducibility analysis, we selected one DICOM clip per participant (n = 124), restricted to gestational ages between 23 and 30 weeks. This interval was chosen based on the intra-observer analysis, which showed optimal reproducibility within this range. Consequently, the inter-observer analysis reflects reproducibility under favorable imaging conditions and may not be representative of the full gestational age range (18–41 weeks). Moreover, as image quality and fetal position can vary substantially in routine clinical practice, the applicability of these findings to less optimal imaging conditions may be limited.

A further limitation is that the inter-observer reproducibility analysis was not conducted under fully independent conditions. Observer two was trained by observer one after the latter had gained additional experience from repeated analyses, and the comparison was based on the second set of measurements of observer one. This may have led to increased alignment between observers and, consequently, an overestimation of inter-observer agreement. Finally, the study was conducted by highly experienced operators, which may limit the generalizability of the findings to routine clinical practice, where varying levels of expertise are encountered.

### Research implications and future directions

This study demonstrates that fSTE yields poor to good reproducibility when the same heart cycle is analyzed within a single DICOM clip. From a clinical perspective, this variability implies that GLS measurements should be interpreted with caution, particularly when small differences are used to guide clinical decisions or to assess longitudinal changes. Before clinical implementation, further research is required to determine how individual steps within the analysis process affect reproducibility. A logical next step would be to evaluate the reproducibility of the heart cycle selection, which is often the first and potentially most variable stage of the workflow. Moreover, the manual delineation and adjustment of the endocardial border in both systole and diastole appear to represent critical sources of variability. Looking ahead, the application of artificial intelligence (AI) for automated selection of the most optimal heart cycle and detection of the endocardial border deserves further investigation, as such automation could minimize observer-dependent variation and improve the consistency and reliability of fSTE measurements.

## Conclusion

Speckle tracking analysis of fetal LV-and RV-GLS demonstrates variable reproducibility after standardizing image and cycle selection. The measurements in the left ventricle showed more reliable reproducibility than those in the right ventricle. Across gestational age groups, descriptively higher ICC values were observed in the mid-gestation groups (23–32 weeks), However, to further define and compare intra- and inter-observer reproducibility in fSTE, additional studies are needed that clearly describe their protocols and use standardized methodologies.

## Supporting information

S1 File Dataset intra-observer reproducibility. (XLSX)

S2 File Dataset interobserver reproducibility. (XLSX)
