# Supplementary material for: Reproducibility of fetal global longitudinal strain measured with speckle tracking echocardiography using a fixed cardiac cycle
Source: PLoS One. 2026 Jul 31;21(7):e0354514. doi: 10.1371/journal.pone.0354514 (PMC13426953; doi:10.1371/journal.pone.0354514)
Supplement: Attachment — Submitted filename: Response to Reviewers_12.06.26.docx [file pone.0354514.s003.docx]

**Responses to reviewers**

**Reviewer 1:**

- The introduction could better highlight the novelty of the study.
  *Answer of the author: Thank you for the relevant suggestion. We adjusted the introduction by adding the novelty of the study (please find this in lines 67 – 71).*
- The methods section would benefit from more detail regarding operator experience and selection criteria.
  *Answer of the author: Thank you for this important remark. We added both the operator's experience and the selection criteria of the clips of the inter-observer reproducibility in the method section (please find this in lines 128-132; 140-141).*
- In the discussion, some sections are slightly repetitive, and the clinical implications of the findings could be more clearly articulated.
  *Answer of the author: Thank you for this important observation. We reviewed the discussion section and removed the repetitive sections where possible. Furthermore, the clinical implications were revised and adjusted (please find this in lines 427-429).*
- Additionally, limitations related to generalizability and software dependence should be expanded.
  *Answer of the author: Thank you for this valuable suggestion. We have expanded the limitations section to further address generalizability (please find this in lines 415-416; 421-423).*

**Reviewer 2:**

(1) Sample size calculation and sample size :

- The authors write that for the sample size calculation they set threshold = 0.75 and expected reliability = 0.60. In the classical approach to planning an ICC reliability study, the expected value should be higher than the minimally acceptable one, not lower. In the present wording, it looks like the study was powered to show that the expected reliability is worse than the preset threshold. This suggests either the parameters were described in reverse, or the calculation itself was done incorrectly.
  *Answer of the author: Thank you for this important observation. In reliability studies using the intraclass correlation coefficient, the expected reliability should indeed be higher than the minimally acceptable (threshold) value, as the statistical test evaluates whether the true ICC exceeds that lower bound. In our original text, the parameters were inadvertently presented in reverse, which may suggest an incorrect study objective. Therefore, we rerun the sample size calculation with the following parameters: Minimum acceptable reliability (ICC) = 0.60; Expected reliability (ICC)= 0.75; Significance level (α):0.05; Power (1 - β): 0.80. Please find the corrected text in line 184.*
- The authors state that the cohort included 124 women, while the inter-observer analysis was performed on 136 independent ultrasound clips, and at the same time, they write they used “only independent data, with one DICOM clip per patient”. These three pieces of information cannot really be reconciled logically.
  *Answer of author: Thank you for carefully noting this inconsistency. The discrepancy arises from the way the dataset was initially handled. The original database contained more than 124 participants, including participants who, e.g., gave birth to a* neonate with a birth weight < 10^th^ percentile or with a congenital or genetic abnormality*. These participants were excluded from the healthy cohort of 124 participants. During preparation of the inter-observer analysis, a subset of ultrasound clips (n = 136) was inadvertently selected, including some clips from participants who were later excluded from the final study cohort. As correctly stated in the Methods, the intended design was to include only one independent clip per patient from the final cohort. Upon identifying this inconsistency, we revised the analysis of the inter-observer reproducibility to include only clips from the 124 eligible participants, ensuring full consistency with the study design (one clip per patient, independent observations, and inclusion restricted to the defined study population). The manuscript has been corrected accordingly, and all analyses now reflect the 124 independent clips from the eligible cohort only (lines 226-229).*
- The authors admit that the clips for the inter-observer analysis came from the gestational age category with the highest reproducibility in the intra-observer subanalysis, and in the end they mostly covered 25 to 30 weeks, with predominance of week 27. This means the inter-observer analysis was not a representative assessment of the whole 18 to 41 week range, but rather an assessment done in a privileged, preselected fragment of the data.
  *Answer of the author: Thank you for this relevant observation. We agree that the inter-observer reproducibility analysis was performed on a subset of the full gestational age range and therefore does not represent the entire GA range from 18 to 41 weeks.*

*As noted in the method section, the clips for the inter-observer analysis were selected from the GA interval (23–30 weeks) that demonstrated the highest reproducibility in the intra-observer analysis. This choice was made deliberately to ensure optimal image quality and tracking reliability when evaluating inter-observer variability, which is known to be sensitive to technical factors such as image quality and fetal position.*

*However, we acknowledge that this approach introduces a selection bias and limits the generalizability of the inter-observer reproducibility results across the full gestational range. The reported ICCs should therefore be interpreted as reflecting reproducibility under relatively favorable conditions, rather than as an estimate applicable to all gestational ages. We added this in the limitation section (lines 410-414).*

(2) Method section

- In both the intra-observer and inter-observer settings, the authors effectively freeze steps 1 and 2, meaning clip selection and cardiac cycle selection. The second intra-observer analysis starts from step 3, and in the inter-observer setting the second observer uses the clip and cycle previously selected by the first observer. So in practice the study does not assess full reproducibility of GLS measurement, but only reproducibility of manual segmentation/contouring and GLS calculation after the clip and cycle had already been fixed. This does not fully invalidate the work, but it needs a more honest naming. The current title and abstract are too broad. This should already be stated in the title, abstract and conclusions.
  *Answer of the author: Thank you for this important comment. We agree that our study design isolates the reproducibility of the post-processing steps, as both clip selection and cardiac cycle selection were fixed in the intra- and inter-observer analyses. We have therefore revised the title (lines 1-2), abstract (lines 20-21; 27-28; 35), and conclusions (line 441) to more accurately reflect that our findings pertain specifically to observer variability in strain analysis under standardized conditions, rather than full measurement reproducibility. We would like to emphasize that we consider this a first step in the evaluation of GLS reproducibility, as variability can arise at multiple stages of the process (image acquisition, clip selection, cycle selection, and post-processing). Our approach allowed us to isolate and better understand variability in the analysis phase.*
- In the Methods, the authors refer to an earlier description of the cohort, but even in this manuskript the basic information is still needed: exact inclusion and exclusion criteria, number of women assessed for eligibilty, number excluded, number of failed or non-analyzable clips, distribution of number of visits per patient, reasons for missing data, and ideally a flow diagram. Without this, it is imposible to judge whether the analyzed clips were a representative subset of the material or rather a selected set of technically best examinations.
  *Answer of author: Thank you for the relevant suggestion. We agree that more information about the study protocol should have been noted in the manuscript to make it more readable. Therefore, we added information about the original study protocol in the method section (please find this in lines 78-87). Furthermore, the main results from the original study are described in the results section (lines 190-195) and in Table 4-5.*

(3) Method: statistical analysis

- The authors correctly criticize the literature for imprecise ICC reporting, but they themself do not provide the most important information, namely which exact ICC model was used. The mere statement that ICC was estimated by LMM does not solve the interpretative problem.
  *Answer of the author: Thank you for this important comment. We agree that precise reporting of the ICC model is essential for proper interpretation. In our analysis, the ICC was derived from a linear mixed-effects model with a random intercept for patient. This approach estimates the proportion of total variance attributable to between-patient variability relative to total variance (between-patient + residual variance). This formulation corresponds to a single-measure, one-way random-effects ICC (ICC(1,1)), reflecting absolute agreement, with the additional inclusion of gestational age as a fixed-effect covariate. The observers were not explicitly modelled as a separate random effect; instead, repeated measurements were treated as nested within patients, implying that measurements are considered exchangeable. We acknowledge that this was not clearly stated in the original manuscript and have now explicitly specified the ICC model and its interpretation to improve clarity and reproducibility. Please find the adjustments in lines 160-163.*
- Also, p-values are attached to tables labeled as ICC tables, but it is not clearly stated whether these are p-values for the ICC test or for the paired t-test related to bias.
  *Answer of the author: Thank you for this important remark. You are correct that the p-values presented in the tables relate to the paired t-test assessing systematic bias between measurements, and not to the ICC estimates themselves. This was not clearly specified in the original version of the manuscript. We have revised the tables and corresponding text to explicitly indicate that these p-values refer to the paired t-test.*
- The whole intra-observer analysis includes 632 clips from 124 patients, so the observations are not independent. The authors use LMM for ICC, which is reasonable, but the Bland-Altman plots and paired t-tests are described as if all clips were treated like independent pairs of measurments. With repeated measurements from the same fetuses, the classical Bland-Altman method may give incorrect limits of agreement if the repeated-measures structure is not taken into account. In this work either a repeated-measures Bland-Altman aproach or a mixed-effects aproach to agreement is needed, or at least a clear discussion of this limitation.
  The most striking aspect of the intra-observer results is not the ICC itself, but the absolute width of the LoA. For the LV, the mean difference is 0.16, but the limits of agreement go to about -11.34 and +11.67, and for the RV about -11.71 to +12.34. With mean GLS values around -21% and -19%, this is a very wide spread. In other words, even with a “moderate” ICC, the clinical interchangebility of single measurements may still be weak.
  *Answer of the author: Thank you for this thoughtful and important comment regarding the interpretation of the Bland–Altman analysis in the presence of repeated measurements. We agree that the intra-observer dataset includes multiple measurements per subject and therefore does not fully meet the independence assumption underlying the classical Bland–Altman method. This may influence the estimated limits of agreement.*

*However, we have chosen to retain the Bland–Altman plots in the intra-observer analysis for the following reasons. First, the primary objective of this study was to assess the reproducibility of the analysis process itself under controlled conditions, specifically by re-analyzing the same cardiac cycle within identical ultrasound clips. In this context, Bland–Altman analysis provides an intuitive and clinically interpretable visualization of the absolute differences between repeated measurements, which cannot be captured by ICC alone.*

*Second, as also highlighted in your comment, the width of the limits of agreement represents an important finding of this study, demonstrating that substantial variability may be present despite moderate ICC values. This has direct implications for the clinical interpretability and interchangeability of individual GLS measurements. Removing the Bland–Altman analysis would limit our ability to communicate this clinically relevant aspect of measurement variability.*

*To address the methodological concern, we have revised the manuscript to explicitly acknowledge that the intra-observer Bland–Altman analysis should be interpreted as descriptive, given the repeated-measures structure of the data (lines 165-168). Furthermore, we emphasize that the primary statistical assessment of reliability is based on ICC derived from linear mixed-effects models, which appropriately account for within-subject correlation.*

(4) Discussion section

- In the discussion the authors themself write that observer one became more precise after her second own measurement and then trained observer two, while the inter-observer analysis was calculated using the second measurement of observer one and the measurement of observer two. This is not a neutral circumstnace. It means the observers were not simply two independent readers, but that the second observer entered the analysis after a stage of harmonisation with the first one. Such a setup may artifically increase agreement and should be treated as a serious limitation, not only as a possible explanation of the resutls.
  *Answer of the author: Thank you for this important and insightful remark. We agree that the inter-observer analysis in our study was not conducted under fully independent conditions. As correctly highlighted, observer two underwent training with observer one to reach consensus on the selection of acceptable clips for fSTE analysis. Prior to this harmonization step, observer two had already received general training in fSTE methodology; however, the subsequent alignment with observer one may have further reduced variability. Furthermore, the inter-observer comparison was based on the second set of measurements of observer one. This design may indeed have led to increased harmonization between observers and could therefore have artificially inflated inter-observer agreement. We acknowledge that this represents an important methodological limitation rather than merely an explanatory factor. Notably, despite this potential for increased agreement, reproducibility remained only moderate, even when using a fixed cardiac cycle, which further underscores the inherent challenges of the technique in the clinic. We have revised the discussion and limitation sections (lines 417-421) to explicitly reflect this and to ensure that the inter-observer reproducibility results are interpreted with appropriate caution.*

- In the discussion there is a sentence saying that “two of the eight studies” did not report 95% CI, but three papers are cited there: [14], [22], and [26].
  *Answer of the author: Thank you for this observation. The text was corrected accordingly, please find this in line 266.*

(5) Conclusion section

- The conclusion about the “highest reproducibility before 32 weeks” is too strong. The authors show descriptively that some ICC values are highest in the 23 to 27 and 28 to 32 week groups. But they did not perform a formal comparison of ICCs between gestational age groups, they did not show an interaction test, and the confidence intervals overlap to a large extent. In additon, the inter-observer analysis was intentionally built on material from exactly this range. In this situation, the statement in the abstract and conclusion that reproducibility is highest before 32 weeks is more narrative then evidence-based. It should be softened to a descriptive observation.
  *Answer of the author: Thank you for this important and well-founded comment. We agree that our original conclusion regarding “highest reproducibility before 32 weeks” was too strong, given the descriptive nature of the analyses. As correctly noted, we did not perform formal statistical comparisons between gestational age groups (e.g., interaction testing), and the confidence intervals overlap substantially. We have therefore revised the conclusion to present this finding as a descriptive observation rather than a definitive statement (lines 443-444).*

(6) Limitations

- In the limitations section the authors write that both intra- and inter-observer reproducibility analyses demonstrated statistically significant ICCs, which is not consistant with the tables, where p-values for intra-observer are 0.499 and 0.221, and for inter-observer LV 0.298.
  *Answer of the author: Thank you for this important observation. We agree that the sentence in the limitations section was not sufficiently precise and may have led to confusion. The p-values presented in the tables correspond to the paired t-test assessing systematic bias between measurements, and not to statistical testing of the ICC estimates. Therefore, the statement that both intra- and inter-observer analyses demonstrated statistically significant ICCs was incorrect.*

*We have revised the limitation section to remove this statement (lines 400-401) and to clarify that only the inter-observer reproducibility of RV-GLS showed a statistically significant mean difference (paired t-test), indicating potential systematic bias between observers.*

- The authors did not consider angle of insonation because previous data supposedly suggested angle-independence. However, their own bibliography includes papers dealing with the effect of angle and frame rate on GLS, and in the discussion they themself admit that previous studies also differed in this respect.
  *Answer of the author: Thank you for this thoughtful comment. We agree that the role of the angle of insonation in GLS measurements remains a point of discussion in the literature. Although some prior studies have suggested relative angle-independence of speckle tracking software, other studies (including those cited) indicate that variation in insonation angle, as well as factors such as frame rate, may influence tracking performance and reproducibility.*

*In our study, the angle of insonation was not explicitly standardized during image acquisition, as our primary aim was to evaluate the reproducibility of the analysis process under controlled conditions using the same cardiac cycle within a given clip. As such, acquisition-related factors were not the main focus of this study design.*

*We have revised the discussion accordingly (lines 385-388).*

(7) Tables

- Table 4 does not match the authors’ own results. The row “Our study” contains numbers and CIs that do not correspond to Tables 1 and 3. In particular, RV inter-observer in Table 4 is given as 0.71 [0.61-0.78], while Table 3 shows 0.625 [0.426-0.777].
  *Answer of the author: Thank you for remarking on this inconsistency. We adjusted Table 4 accordingly.*

(8) Data statement

- The data statement says: “All relevant data are within the manuscript and its Supporting Information files”, but in the submitted draft there are no actual source data allowing the reader to reproduce the reproducibility analyses for 632 clips and 3 reading series. If the relevant dataset is supposed to be in the supplementary material, it should be actually attached and described. Otherwise the statement is incomplete or misleading.
  *Answer of the author: Thank you for pointing this out. We acknowledge that in the originally submitted draft, the data underlying the reproducibility analyses were not included, despite the statement indicating that all relevant data were available in the manuscript and Supporting Information. We have now addressed this issue by uploading the complete dataset associated with the 632 clips and the three reading series as supplementary material. The files have been clearly labeled and described to ensure that readers can access and use them to reproduce the analyses presented in the manuscript.*
