# Supplementary material for: Reproducibility of fetal global longitudinal strain measured with speckle tracking echocardiography using a fixed cardiac cycle
Source: PLoS One. 2026 Jul 31;21(7):e0354514. doi: 10.1371/journal.pone.0354514 (PMC13426953; doi:10.1371/journal.pone.0354514)
Supplement: Peer review history

# Peer review: Decision Letter 0

**DOI:** 10.1371/journal.pone.0354514.r001

**Authors:** Francesca Crovetto

7 May 2026

PONE-D-26-05691

Reproducibility of fetal global longitudinal strain measured with speckle tracking echocardiography

PLOS One

Dear Dr. Meireson,

Thank you for submitting your manuscript to PLOS ONE. After careful consideration, we feel that it has merit but does not fully meet PLOS ONE’s publication criteria as it currently stands. Therefore, we invite you to submit a revised version of the manuscript that addresses the points raised during the review process.

Please submit your revised manuscript by Jun 21 2026 11:59PM. If you will need more time than this to complete your revisions, please reply to this message or contact the journal office at plosone@plos.org. When you're ready to submit your revision, log on to https://www.editorialmanager.com/pone/ and select the 'Submissions Needing Revision' folder to locate your manuscript file.

Please include the following items when submitting your revised manuscript:

- A letter that responds to each point raised by the academic editor and reviewer(s). You should upload this letter as a separate file labeled 'Response to Reviewers'.
- A marked-up copy of your manuscript that highlights changes made to the original version. You should upload this as a separate file labeled 'Revised Manuscript with Track Changes'.
- An unmarked version of your revised paper without tracked changes. You should upload this as a separate file labeled 'Manuscript'.

If you would like to make changes to your financial disclosure, please include your updated statement in your cover letter. Guidelines for resubmitting your figure files are available below the reviewer comments at the end of this letter.

If applicable, we recommend that you deposit your laboratory protocols in protocols.io to enhance the reproducibility of your results. Protocols.io assigns your protocol its own identifier (DOI) so that it can be cited independently in the future. For instructions see: https://journals.plos.org/plosone/s/submission-guidelines#loc-laboratory-protocols. Additionally, PLOS ONE offers an option for publishing peer-reviewed Lab Protocol articles, which describe protocols hosted on protocols.io. Read more information on sharing protocols at https://plos.org/protocols?utm_medium=editorial-email&utm_source=authorletters&utm_campaign=protocols.

As the corresponding author, your ORCID iD is verified in the submission system and will appear in the published article. PLOS supports the use of ORCID, and we encourage all coauthors to register for an ORCID iD and use it as well. Please encourage your coauthors to verify their ORCID iD within the submission system before final acceptance, as unverified ORCID iDs will not appear in the published article. *Only*  the individual author can complete the verification step; PLOS staff *cannot*  verify ORCID iDs on behalf of authors.

We look forward to receiving your revised manuscript.

Kind regards,

Francesca Crovetto

Academic Editor

PLOS One

Journal Requirements:

When submitting your revision, we need you to address these additional requirements.

1. Please ensure that your manuscript meets PLOS ONE's style requirements, including those for file naming. The PLOS ONE style templates can be found at https://journals.plos.org/plosone/s/file?id=wjVg/PLOSOne_formatting_sample_main_body.pdf and https://journals.plos.org/plosone/s/file?id=ba62/PLOSOne_formatting_sample_title_authors_affiliations.pdf

2. We note that your Data Availability Statement is currently as follows: All relevant data are within the manuscript and its Supporting Information files.

Please confirm at this time whether or not your submission contains all raw data required to replicate the results of your study. Authors must share the “minimal data set” for their submission. PLOS defines the minimal data set to consist of the data required to replicate all study findings reported in the article, as well as related metadata and methods (https://journals.plos.org/plosone/s/data-availability#loc-minimal-data-set-definition).

For example, authors should submit the following data:

- The values behind the means, standard deviations and other measures reported;

- The values used to build graphs;

- The points extracted from images for analysis.

Authors do not need to submit their entire data set if only a portion of the data was used in the reported study.

If your submission does not contain these data, please either upload them as Supporting Information files or deposit them to a stable, public repository and provide us with the relevant URLs, DOIs, or accession numbers. For a list of recommended repositories, please see https://journals.plos.org/plosone/s/recommended-repositories.

If there are ethical or legal restrictions on sharing a de-identified data set, please explain them in detail (e.g., data contain potentially sensitive information, data are owned by a third-party organization, etc.) and who has imposed them (e.g., an ethics committee). Please also provide contact information for a data access committee, ethics committee, or other institutional body to which data requests may be sent. If data are owned by a third party, please indicate how others may request data access.

3. If the reviewer comments include a recommendation to cite specific previously published works, please review and evaluate these publications to determine whether they are relevant and should be cited. There is no requirement to cite these works unless the editor has indicated otherwise.

[Note: HTML markup is below. Please do not edit.]

Reviewers' comments:

Reviewer's Responses to Questions

**Comments to the Author**

1. Is the manuscript technically sound, and do the data support the conclusions?

The manuscript must describe a technically sound piece of scientific research with data that supports the conclusions. Experiments must have been conducted rigorously, with appropriate controls, replication, and sample sizes. The conclusions must be drawn appropriately based on the data presented.

Reviewer #1: Yes

Reviewer #2: Partly

**********

2. Has the statistical analysis been performed appropriately and rigorously?

Reviewer #1: Yes

Reviewer #2: Yes

**********

3. Have the authors made all data underlying the findings in their manuscript fully available?

The PLOS Data policy requires authors to make all data underlying the findings described in their manuscript fully available without restriction, with rare exception (please refer to the Data Availability Statement in the manuscript PDF file). The data should be provided as part of the manuscript or its supporting information, or deposited to a public repository. For example, in addition to summary statistics, the data points behind means, medians and variance measures should be available. If there are restrictions on publicly sharing data—e.g. participant privacy or use of data from a third party—those must be specified.

Reviewer #1: Yes

Reviewer #2: No

**********

4. Is the manuscript presented in an intelligible fashion and written in standard English?

PLOS ONE does not copyedit accepted manuscripts, so the language in submitted articles must be clear, correct, and unambiguous. Any typographical or grammatical errors should be corrected at revision, so please note any specific errors here.

Reviewer #1: Yes

Reviewer #2: Yes

**********

5. Review Comments to the Author

Please use the space provided to explain your answers to the questions above. You may also include additional comments for the author, including concerns about dual publication, research ethics, or publication ethics. (Please upload your review as an attachment if it exceeds 20,000 characters)

Reviewer #1: I have carefully reviewed the manuscript entitled “Reproducibility of fetal global longitudinal strain measured with speckle tracking echocardiography” . Overall, I find it to be a well-conducted and relevant study addressing an important limitation in fetal cardiology, namely the variability of fSTE measurements that currently limits clinical implementation.

The manuscript is generally well structured, with a clear objective and a solid methodological approach. I particularly appreciate the use of a standardized protocol and the decision to analyze the same heart cycle within a single DICOM clip, which represents a meaningful methodological strength. The relatively large sample size further enhances the reliability of the findings.

The results are clearly presented and show that reproducibility is variable, with better performance in the left ventricle and before 32 weeks of gestation. The finding of higher inter-observer than intra-observer reproducibility is interesting and deserves further emphasis. What I found particularly valuable is the clarity with which the methodological steps are described, especially the detailed explanation of the fSTE analysis workflow. This makes the study easy to follow and gives confidence in the reproducibility of the approach.

That said, a few aspects could be improved. The introduction could better highlight the novelty of the study, and the methods section would benefit from more detail regarding operator experience and selection criteria. In the discussion, some sections are slightly repetitive, and the clinical implications of the findings could be more clearly articulated. Additionally, limitations related to generalizability and software dependence should be expanded.

The manuscript includes a limited number of citations from the authors’ previous work, which appear appropriate given the continuity of the research and the use of an established cohort and methodology.

In conclusion, this is a valuable and methodologically sound study. With minor to moderate revisions, particularly to improve clarity and strengthen the discussion, the manuscript would be suitable for publication.

Reviewer #2: The authors make a pretty reasonable atempt to separate the soruces of variability in fSTE by analyzing the same cardiac cycle in the same DICOM clip, which by itself is valuable and honestly more precise then many earlier studies.

My comments on the manuskript:

The authors state that the cohort included 124 women, while the inter-observer analysis was performed on 136 independent ultrasound clips, and at the same time they write they used “only independent data, with one DICOM clip per patient”. These three pieces of information cannot really be reconciled logicaly.

The authors write that for the sample size calculation they set threshold = 0.75 and expected reliability = 0.60. In the classical aproach to planning an ICC reliability study, the expected value should be higher then the minimally acceptable one, not lower. In the present wording it looks like the study was powered to show that the expected reliabilty is worse than the preset threshold. This suggests either the parameters were described in reverse, or the calculation itself was done incorretly.

The authors admit that the clips for the inter-observer analysis came from the gestational age category with the highest reproducibility in the intra-observer subanalysis, and in the end they mostly covered 25 to 30 weeks, with predominance of week 27. This means the inter-observer analysis was not a representative assesment of the whole 18 to 41 week range, but rather an assesment done in a priviliged, preselected fragment of the data.

In both the intra-observer and inter-observer settings, the authors efectively freeze steps 1 and 2, meaning clip selection and cardiac cycle selection. The second intra-observer analysis starts from step 3, and in the inter-observer setting the second observer uses the clip and cycle previously selected by the first observer. So in practice the study does not assess full reproducibility of GLS measurement, but only reproducibility of manual segmentation/contouring and GLS calculation after the clip and cycle had already been fixed. This does not fully invalidate the work, but it needs a more honest naming. The current title and abstract are too broad. This should already be stated in the title, abstract and conclusions.

In the discussion the authors themself write that observer one became more precise after her second own measurement and then trained observer two, while the inter-observer analysis was calculated using the second measurement of observer one and the measurement of observer two. This is not a neutral circumstnace. It means the observers were not simply two independent readers, but that the second observer entered the analysis after a stage of harmonisation with the first one. Such a setup may artifically increase agreement and should be treated as a serious limitation, not only as a possible explanation of the resutls.

The authors correctly criticize the literature for imprecise ICC reporting, but they themself do not provide the most important information, namely which exact ICC model was used. The mere statement that ICC was estimated by LMM does not solve the interpretative problem. Also, p-values are attached to tables labeled as ICC tables, but it is not clearly stated whether these are p-values for the ICC test or for the paired t-test related to bias.

The whole intra-observer analysis includes 632 clips from 124 patients, so the observations are not independent. The authors use LMM for ICC, which is reasonable, but the Bland-Altman plots and paired t-tests are described as if all clips were treated like independent pairs of measurments. With repeated measurements from the same fetuses, the classical Bland-Altman method may give incorrect limits of agreement if the repeated-measures structure is not taken into account. In this work either a repeated-measures Bland-Altman aproach or a mixed-effects aproach to agreement is needed, or at least a clear discussion of this limitation.

The most striking aspect of the intra-observer results is not the ICC itself, but the absolute width of the LoA. For the LV, the mean difference is 0.16, but the limits of agreement go to about -11.34 and +11.67, and for the RV about -11.71 to +12.34. With mean GLS values around -21% and -19%, this is a very wide spread. In other words, even with a “moderate” ICC, the clinical interchangebility of single measurements may still be weak.

The conclusion about the “highest reproducibility before 32 weeks” is too strong. The authors show descriptively that some ICC values are highest in the 23 to 27 and 28 to 32 week groups. But they did not perform a formal comparison of ICCs between gestational age groups, they did not show an interaction test, and the confidence intervals overlap to a large extent. In additon, the inter-observer analysis was intentionally built on material from exactly this range. In this situation, the statement in the abstract and conclusion that reproducibility is highest before 32 weeks is more narrative then evidence-based. It should be softened to a descriptive observation.

In the limitations section the authors write that both intra- and inter-observer reproducibility analyses demonstrated statistically significant ICCs, which is not consistant with the tables, where p-values for intra-observer are 0.499 and 0.221, and for inter-observer LV 0.298.

In the discussion there is a sentence saying that “two of the eight studies” did not report 95% CI, but three papers are cited there: [14], [22], and [26].

Table 4 does not match the authors’ own results. The row “Our study” contains numbers and CIs that do not correspond to Tables 1 and 3. In particular, RV inter-observer in Table 4 is given as 0.71 [0.61-0.78], while Table 3 shows 0.625 [0.426-0.777].

In the Methods, the authors refer to an earlier description of the cohort, but even in this manuskript the basic information is still needed: exact inclusion and exclusion criteria, number of women assessed for eligibilty, number excluded, number of failed or non-analyzable clips, distribution of number of visits per patient, reasons for missing data, and ideally a flow diagram. Without this, it is imposible to judge whether the analyzed clips were a representative subset of the material or rather a selected set of technically best examinations.

The data statement says: “All relevant data are within the manuscript and its Supporting Information files”, but in the submitted draft there are no actual source data allowing the reader to reproduce the reproducibility analyses for 632 clips and 3 reading series. If the relevant dataset is supposed to be in the supplementary material, it should be actually attached and described. Otherwise the statement is incomplete or misleaading.

The authors did not consider angle of insonation because previous data supposedly suggested angle-independence. However, their own bibliography includes papers dealing with the effect of angle and frame rate on GLS, and in the discussion they themself admit that previous studies also differed in this respect.

Overall, I think the manuskript may become suitable for publication, but only after an apropriate major revision.

**********

6. PLOS authors have the option to publish the peer review history of their article (what does this mean?). If published, this will include your full peer review and any attached files.

If you choose “no”, your identity will remain anonymous but your review may still be made public.

**Do you want your identity to be public for this peer review?**  For information about this choice, including consent withdrawal, please see our Privacy Policy.

Reviewer #1: **Yes:**  Cerghit-Paler Andreea

Reviewer #2: No

**********

[NOTE: If reviewer comments were submitted as an attachment file, they will be attached to this email and accessible via the submission site. Please log into your account, locate the manuscript record, and check for the action link "View Attachments". If this link does not appear, there are no attachment files.]

To ensure your figures meet our technical requirements, please review our figure guidelines: https://journals.plos.org/plosone/s/figures

You may also use PLOS’s free figure tool, NAAS, to help you prepare publication quality figures: https://journals.plos.org/plosone/s/figures#loc-tools-for-figure-preparation.

NAAS will assess whether your figures meet our technical requirements by comparing each figure against our figure specifications.

# Author comment: Author response to Decision Letter 1

**DOI:** 10.1371/journal.pone.0354514.r002

16 Jun 2026

Ghent University

Human structure and repair

Corneel Heymanslaan 10

9000 Gent, Belgium

Dear Francesca Crovetto,

On behalf of all the authors, I hereby kindly submit our revised manuscript entitled “Reproducibility of fetal global longitudinal strain measured with speckle tracking echocardiography using a fixed cardiac cycle” (reference number: PONE-D-26-05691).

We appreciate the opportunity to revise our manuscript and are grateful to you and the reviewers for the constructive and detailed feedback. We have carefully addressed all comments and believe that the revisions have strengthened the manuscript.

Please find attached a point-by-point response to the reviewers’ comments. All changes made in the manuscript are also clearly indicated in the track-changes version.

Please address all correspondence concerning this manuscript to: eline.meireson@ugent.be

Thank you for considering our manuscript.

With kind regards,

Eline Meireson, PhD student

Chantelle de Vet, MD, PhD, consultant physician

Judith O.E.H. van Laar, MD, PhD, consultant obstetrician, associated professor TU/e

Phaedra Mussche, master student biomedical science

Prof. Kristien Roelens, MD, PhD, consultant obstetrician

Noortje H. M. van Oostrum, MD, PhD, consultant obstetrician

Attachment Submitted filename: Response to Reviewers_12.06.26.docx

# Editor report: Decision Letter 1

**DOI:** 10.1371/journal.pone.0354514.r003

**Authors:** Francesca Crovetto

10 Jul 2026

Reproducibility of fetal global longitudinal strain measured with speckle tracking echocardiography using a fixed cardiac cycle

PONE-D-26-05691R1

Dear Dr. Meireson

We’re pleased to inform you that your manuscript has been judged scientifically suitable for publication and will be formally accepted for publication once it meets all outstanding technical requirements.

Within one week, you’ll receive an e-mail detailing the required amendments. When these have been addressed, you’ll receive a formal acceptance letter and your manuscript will be scheduled for publication.

An invoice will be generated when your article is formally accepted. Please note, if your institution has a publishing partnership with PLOS and your article meets the relevant criteria, all or part of your publication costs will be covered. Please make sure your user information is up-to-date by logging into Editorial Manager at Editorial Manager® and clicking the ‘Update My Information' link at the top of the page. For questions related to billing, please contact billing support.

If your institution or institutions have a press office, please notify them about your upcoming paper to help maximize its impact. If they’ll be preparing press materials, please inform our press team as soon as possible -- no later than 48 hours after receiving the formal acceptance. Your manuscript will remain under strict press embargo until 2 pm Eastern Time on the date of publication. For more information, please contact onepress@plos.org.

Kind regards,

Francesca Crovetto

Academic Editor

PLOS One

# Editor report: Acceptance letter

**DOI:** 10.1371/journal.pone.0354514.r004

**Authors:** Francesca Crovetto

PONE-D-26-05691R1

PLOS One

Dear Dr. Meireson,

I'm pleased to inform you that your manuscript has been deemed suitable for publication in PLOS One. Congratulations! Your manuscript is now being handed over to our production team.

At this stage, our production department will prepare your paper for publication. This includes ensuring the following:

* All references, tables, and figures are properly cited

* All relevant supporting information is included in the manuscript submission,

* There are no issues that prevent the paper from being properly typeset

You will receive further instructions from the production team, including instructions on how to review your proof when it is ready. Please keep in mind that we are working through a large volume of accepted articles, so please give us a few days to review your paper and let you know the next and final steps.

Lastly, if your institution or institutions have a press office, please let them know about your upcoming paper now to help maximize its impact. If they'll be preparing press materials, please inform our press team within the next 48 hours. Your manuscript will remain under strict press embargo until 2 pm Eastern Time on the date of publication. For more information, please contact onepress@plos.org.

You will receive an invoice from PLOS for your publication fee after your manuscript has reached the completed accept phase. If you receive an email requesting payment before acceptance or for any other service, this may be a phishing scheme. Learn how to identify phishing emails and protect your accounts at https://explore.plos.org/phishing.

If we can help with anything else, please email us at customercare@plos.org.

Thank you for submitting your work to PLOS ONE and supporting open access.

Kind regards,

PLOS ONE Editorial Office Staff

on behalf of

Dr. Francesca Crovetto

Academic Editor

PLOS One
